# Influence of Cultural Norms on Formal Service Engagement Among Survivors of Intimate Partner Violence: A Qualitative Meta-synthesis

**DOI:** 10.1177/15248380231162971

**Published:** 2023-04-19

**Authors:** Jane Green, Lata Satyen, John W. Toumbourou

**Affiliations:** 1 Deakin University, Melbourne, Australia

**Keywords:** intimate partner violence, violence against women, cultural norms, service engagement, help-seeking, formal services, family violence, domestic violence

## Abstract

For victim-survivors of intimate partner violence (IPV), receiving help from formal services such as specialist family violence, health, or criminal justice services can be critical for their safety and well-being. Previous research has found cross-cultural differences in the rates of help-seeking behavior, with women from non-Anglo-Saxon communities less likely to seek formal help than Anglo-Saxon populations. This qualitative meta-synthesis has integrated qualitative evidence to examine the relationship between specific cultural norms and formal service engagement for female victim-survivors of IPV from non-Anglo-Saxon communities. A comprehensive search of seven databases was conducted for peer-reviewed articles published between 1985 to May 2021, in addition to searching gray literature. Thirty-five articles met the criteria for inclusion, representing 1,286 participants from 20 cultural groups. Based on a thematic synthesis approach, five key themes that captured specific cultural norms that influence formal service engagement were identified: (1) gender roles and social expectations, (2) community recognition and acceptance of abuse, (3) honor-based society, (4) the role of religion, and (5) cultural beliefs and attitudes toward formal services. These findings have important implications for responses to family violence, particularly concerning family violence education for non-Anglo-Saxon ethnically diverse communities and best-practice strategies to improve the cultural relevancy of formal service providers.

## Background

Intimate partner violence (IPV) refers to a set of behaviors conducted by a former or current intimate partner that cause physical, sexual, or psychological harm and is one of the most common forms of violence against women (World Health Organization [WHO], 2021). IPV affects almost one in three women globally, with one in seven women experiencing IPV in the last 12 months alone (WHO, 2021). The impacts of the COVID-19 global pandemic have exacerbated rates of violence against women, with 45% of women reporting direct or indirect exposure to violence since the start of the pandemic (United Nations Women, 2021).

The lifetime prevalence rate of reported IPV varies between countries from 13 to 61% of ever-partnered women (García-Moreno et al., 2005); however, there is a growing body of literature that suggests women from non-Anglo-Saxon communities may be at greater risk of IPV victimization (Capaldi et al., 2012; García-Moreno et al., 2005; Raj & Silverman, 2002). For the purposes of this research, the term non-Anglo Saxon is used to capture all ethnic groups other than Anglo English-speaking majority groups, rather than the term culturally diverse (Sawrikar & Katz, 2008), to ensure that non-Anglo-Saxon groups living as a cultural majority are also captured. While the term non-Anglo encompasses a broad range of cultures and ethnicities, it is acknowledged that these individuals and groups will have unique and diverse experiences and challenges as well as commonalities in their experience of IPV and the cultural norms that influence service engagement (Kasturirangan & Williams, 2003).

The lowest prevalence rates of IPV occur in Europe (16–23%) while the highest rates range from 33 to 51% in the regions of Oceania, Southern Asia, and Sub-Saharan Africa (García-Moreno et al., 2005; WHO, 2021). Such significant differences across regions indicate that macrosocial factors may be playing a role; these include lower rates of gender equality, adherence to rigid gender roles, and cultural norms that ascribe a higher status to men than women (García-Moreno et al., 2005; Zapata-Calvente et al., 2019). Cultural norms are defined as the shared cultural understandings, values, and beliefs about how society “should” function and delineate appropriate behavior within a culture (Durlauf & Blume, 2008; Garcia-Moreno et al., 2015). While cultural norms bring harmony in communities, they can also contribute to practices that materialize as specific forms of IPV, such as forced marriage and dowry-related violence that place women from non-Anglo-Saxon communities at greater risk of victimization (Raj & Silverman, 2002).

IPV can have significant and long-term impacts on women’s physical, mental, sexual, and reproductive health and can have adverse effects on their children, including cognitive and behavioral issues (WHO, 2021). Existing literature indicates that the impacts of IPV victimization may be lessened by women’s help-seeking behaviors (Meyer, 2010), which are adaptive coping strategies involving the disclosure of personal experience to gain assistance (Mays et al., 1996). Help-seeking strategies are commonly grouped into either informal or formal sources of support (Cho et al., 2020). Informal support includes assistance from family, neighbors, and friends, while formal support includes social, legal, justice, health, and specialist family violence services (Coker et al., 2000). For this research, religious leaders and institutions are considered informal sources, acknowledging however, their increasingly important role in family violence education and prevention (Hyman et al., 2006).

Engagement with formal support services can provide women with the resources required to enhance understanding of violence, improve decision making, and enhance coping skills (Bennett et al., 2004; Ravi et al., 2021). Successful service engagement can improve outcomes for women by implementing harm reduction strategies that reduce negative post-trauma outcomes and improve general well-being (Meyer, 2010). Formal services can be particularly important in providing risk management, safety planning, pathways to recovery, and supporting women to permanently end violent relationships, due to the longevity and stability of supports (Liang et al., 2005), whereas informal support may diminish over time (Coker et al., 2000; Kaukinen, 2004). Despite the known benefits of formal service engagement, rates of formal help-seeking are low (Robinson et al., 2020).

Several studies have demonstrated lower rates of help-seeking among victim-survivors from non-Anglo-Saxon communities compared to Anglo-Saxon populations (Cho et al., 2020; Flicker et al., 2011; Satyen et al., 2018). In 2005, the WHO multi-country study found that between 55% and 95% of women who had been physically abused by a partner never sought help from formal services or people in positions of authority: women reported this to be a result of believing that the violence was normal or not serious (García-Moreno et al., 2005). However, observations of help-seeking behaviors among non-Anglo-Saxon communities are varied. A systematic review of cross-cultural differences in help-seeking by Satyen et al. (2019) found that although Latina/Hispanic and African American women were less likely to engage in formal help-seeking than Caucasian women overall, they were more likely to utilize primary health care and law enforcement services. These findings indicate that differences in help-seeking may be better understood in the context of the types of formal support sought, rather than the frequency (Flicker et al., 2011).

Poor engagement among non-Anglo-Saxon communities may be a result of individual, practical, and cultural barriers that prevent help-seeking (Forbes-Mewett & McCulloch, 2016). These barriers include limited language skills, social isolation, financial dependence on spouses, lack of knowledge of service availability, lack of culturally relevant services, and cultural norms that create a shame and stigma in seeking help (Forbes-Mewett & McCulloch, 2016; Satyen et al., 2018; Satyen et al., 2021). Though these barriers are not unique to women from non-Anglo-Saxon communities, these women are at greater risk due to intersecting inequalities of race, gender, and socioeconomic status that influence women’s help-seeking behavior, as discussed in the intersectionality theory (Crenshaw, 1991; State of Victoria, 2016). The help-seeking theory proposed by Liang et al. (2005) suggests that help-seeking consists of three stages: (1) problem recognition and definition, (2) the decision to seek help, and (3) the selection of a help provider. All three stages are influenced by individual, interpersonal, and sociocultural influences that shape how women perceive the abuse and their role in causing it. Only when women acknowledge the violence as a problem and recognize that they are not at fault, will they begin to recognize the need for support (Liang et al., 2005).

Though literature that considers the specific cultural norms that influence help-seeking is limited, several studies have indicated that gender roles that view women as responsible for family unity place an expectation on women to tolerate the abuse and place the needs of the family ahead of their own (Bui & Morash, 2007; Magnussen et al., 2011; Satyen, 2021; Satyen et al., 2020). These norms reflect a system of female subordination and passivity which silence women and create an unwillingness to seek help due to a fear of repercussions from the perpetrator and the community (Hunnicutt, 2009; Satyen et al., 2021). Formal help-seeking is seen as particularly stigmatizing among non-Anglo-Saxon communities. Services perceived to be “outside” of the community are viewed as having misaligned interests and a potential threat to family harmony: seeking their support could disgrace not only the individual but the broader family and community (Kaukinen & DeMaris, 2009; Kim & Hogge, 2015). As a result, women are more inclined to rely on their own coping mechanisms or informal sources of support (Beaulaurier et al., 2007; Liang et al., 2005).

The influences of cultural norms for women from non-Anglo-Saxon communities are important to understand given the increasing globalization and expansion of multicultural societies across the globe (Gibson, 2020). In 2020, the *World Migration Report* found a continuing upward trend in international migrants, estimating that there were 281 million international migrants in 2020, equivalent to 3.6% of the global population (Gibson, 2020). As rates of cultural diversity increase globally, service providers need to be prepared to respond to the needs of women from multicultural groups and respect their desire to maintain cultural values.

At present, there are no studies that have synthesized qualitative evidence of the influence of cultural norms on formal service engagement specifically. The existing body of literature largely considers the experiences of specific groups, rather than the common experiences across a range of non-Anglo-Saxon groups. Given the known benefits of formal services, an enhanced understanding of the specific cultural norms which influence formal service engagement is critical to provide insight into best practice strategies for formal service providers and improve outcomes for non-Anglo-Saxon communities. This meta-synthesis aims to integrate qualitative evidence to examine the relationship between specific cultural norms and formal service engagement for female victims of IPV in non-Anglo-Saxon communities.

## Method

### Search Strategy

A comprehensive search was performed for articles published from 1985 to May 2022 in the following databases: EMBASE, Medline, PsycINFO, ERIC, CINAHL, GlobalHealth, SocINDEX, PsycExtra, in addition to hand-searching references cited in the included studies. Gray literature was searched as the meta-synthesis includes qualitative rather than quantitative studies (Noyes et al., 2021). APA PsycExtra was selected as it is the leading resource for information in the behavioral and social sciences and is updated bi-weekly.

The comprehensive search strategy (Appendix A) was created in consultation with a library scientist and utilized different combinations of keywords for the following concepts: “cultural norms,” “service engagement,” “intimate partner violence,” and “cultural diversity.”

### Study Selection

#### Inclusion and exclusion criteria

Studies met the inclusion criteria where they: demonstrated qualitative methodology; identified the population of interest as female victims of IPV from non-Anglo-Saxon communities; included female victims of IPV from non-Anglo or community members or service providers that engage with female victims of IPV from non-Anglo-Saxon communities; were published in English; were published between 1985 and 2022 and were peer-reviewed. Studies that did not meet the above criteria, did not distinguish formal support from informal support, did not identify specific cultural norms, and did not contain direct quotes from participants, were excluded. As gray literature captures materials outside of the peer-review process, the requirement for studies to be peer-reviewed was removed for this search. For this research, cultural diversity did not include women who identify as Indigenous, as, discussed by Pham et al. (2021), the distinct and specific needs of Indigenous women should be considered separately.

### Study Screening

**Figure 1.**
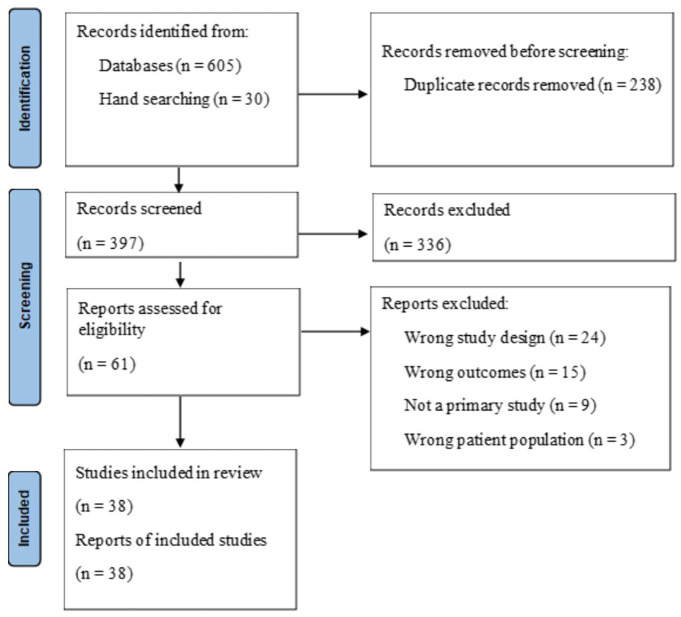
Preferred Reporting Items for Systematic Reviews and Meta-Analyses flow diagram for study selection. *Source*. Adapted from Page et al. (2021).

The reference management system, Covidence was used throughout the review management process to provide an audit trail for decision making. Titles and abstracts from the initial search were imported into Covidence, where duplicates were removed, and the title and abstracts of the remaining articles were subsequently reviewed by a single reviewer (JG). Following the title and abstract screening, JG assessed the full text of the selected articles to determine their relevance and inclusion. A second reviewer (LS) voted for inclusion or exclusion where there was uncertainty. Both reviewers, in addition to an independent third reviewer (JT), reviewed the included articles to confirm that criteria were met and to assess the quality of each of the articles. The results of this study screening are included in Figure 1.

### Methodological Quality Appraisal

Included studies were appraised for methodological quality using the Critical Appraisal Skills Programme (CASP) Qualitative Checklist (CASP, 2019). A value of “Yes,” “No,” or “Can’t Determine” was assigned to each requirement. To add rigor, an additional assessment of the methodological quality was included using the GRADE CerQual method (Munthe-Kaas et al., 2018). Using this method, each study was assigned a rating of “no or very minor concerns,” “minor concerns,” “moderate concerns,” or “serious concerns.” Three reviewers (JG, JT, and LS) assessed the quality of the studies. Results of this appraisal are captured in Appendix B.

### Data Extraction

Data and supporting information from the included studies were extracted into a template developed for this review. Supporting information includes characteristics of the study such as the study objective, participant information, study design, location, and analysis method. A summary of the methodological characteristics is included in Appendix C.

### Data Analysis

The included articles were imported into the software program NVivo for analysis of the data. Thomas and Harden’s (2008) thematic synthesis approach was utilized to analyze patterns across the findings and results sections of the included articles as detailed in the ensuing sections.

#### Stages 1 and 2: Coding text and developing descriptive themes

The first step in the thematic synthesis involved inductive coding of each sentence of the results, staying as close to the language of the raw data as possible. Each sentence was assigned at least one code. This process allowed for concepts to be translated from one study to another and created a total of 542 codes. Codes were checked for consistency of interpretation and duplication and organized into a hierarchical tree structure by two reviewers (LS and JG). Where required, new codes were created to capture the meaning of the groups of initial codes. A total of 16 themes were created, each containing multiple layers of subthemes.

#### Stage 3: Generating analytical themes

Two reviewers (LS and JG) discussed the 16 themes in the context of the research question. Through this discussion, more analytical themes were generated. In some cases, the original descriptive theme remained; however, in most instances new analytical themes were generated subsuming descriptive themes. This process was iterative until each theme responded to the research question and adequately captured all the descriptive themes. This process resulted in a total of five analytical themes.

## Results

A total of 38 studies published between 1996 and 2021 were included in the results. Figure 1 (Page et al., 2021) depicts the selection of the studies following the Preferred Reporting Items for Systematic Reviews and Meta-Analyses guidelines. Studies represented over 20 cultural groups from a range of countries and considered engagement with a range of formal services including health, mental health, criminal justice, specialist family violence, and counseling services.

### Common Themes

Thematic synthesis of the included studies led to the development of five key themes, as captured in Table 1. The five themes are not mutually exclusive and overlap in their relationship to formal help-seeking. A detailed summary of the themes is provided in Appendix D (Table 2).

**Table 1.** Critical Findings of This Study.

	Five Identified Themes Derived From the Studies
1.	Gender roles and social expectations: Beliefs about the role of females, marital obligations and the expectation for women to tolerate abuse were common themes across the studies that influenced women’s service engagement.
2.	Community recognition and acceptance of abuse: The normalization and privatization of abuse was a reoccurring theme across many studies. However, many studies identified that acceptance of abuse was often moderated by the severity.
3.	Honor-based society: Women’s willingness to suffer silently to protect the community reputation, particularly from “outside” intervention, was commonly described. However, some studies identified that communities also provided protection from the perpetrator, with communities holding the perpetrator to account.
4.	Role of religion: Women described turning to religious practices and leaders as a means of coping and for hope. At times, this support was beneficial; however, it often resulted in the reinforcement of norms that silence women and normalize abuse.
5.	Cultural beliefs and attitudes toward formal services: Women described a reluctance to engage with services due to a lack of trust and lack of cultural understanding from services.

**Table 2.** Implications for Practice, Policy, and Research.

1.	The findings of this research indicate a need for greater focus on prevention and early intervention strategies at improving family violence education and awareness of IPV and the support available to culturally diverse communities.
2.	Given the pervasive distrust in services, providers should focus engagement efforts on building trust with culturally diverse communities and seeking input from the community to build cultural humility and relevance.
3.	Cultural norms have a significant influence on women’s recognition, perception, and response to abuse and as such, service providers should seek to understand and consider the influence of cultural norms when attempting to engage with women from culturally diverse communities.
4.	Future research should utilize co-design methodology to develop, in partnership with culturally diverse communities, best-practice strategies for engagement and evaluate the effectiveness of these strategies.

*Note*. IPV = intimate partner violence.

#### Theme 1: Gender roles and social expectations

This theme describes the internalized norms and values that delineate how males and females are expected to behave within their culture. Patriarchal social structures foster an environment in which gender roles view women as responsible for maintaining family unity, above and beyond their safety. The impact of these patriarchal beliefs and gender roles are detailed in the following section.

##### Patriarchal beliefs

Twenty-one studies (specified in Appendix D) discussed the influence of patriarchal beliefs on IPV. Women described how exposure to, and internalization of intergenerational gender norms taught them to be submissive and subservient to male dominance, coined as “machismo” in Latino cultures (Lewis et al., 2005). In the Tse (2007) study, an Asian migrant victim living in New Zealand described that “Men are brought up to be aggressive . . . Women are brought up to service men, to be obedient, to be submissive” (p. 176). These norms placed an expectation on women to defer to male authority and in some instances, women were considered the property of men. Participants also described expectations for men to be providers for the household and women to be responsible for maintaining family harmony. The expectation for women to nurture the family resulted in them being blamed for the occurrence of violence, as elucidated in the following quote from a Mexican migrant victim living in Los Angeles:

Sometimes, in my way of thinking, [women] are at fault. He comes home tired from work, and the only thing that he says is, “Stop bothering me.” But she continues, and that’s why sometimes a man gets to the point of striking. (Acevedo, 2000, p. 259).

As a result of these gender role expectations, women described themselves as being dependent on men, particularly financially, and lacking the self-esteem and autonomy required to seek help. Women were also further discouraged where they believed services mirrored these patriarchal structures.

##### Marital roles and obligations

Participants across 25 studies (see Appendix D) described expectations of women to be a “good wife,” further exacerbating gender norms that view women as subservient. Participants described that marriage allowed men to take ownership of women and their bodies: “your body belongs to him until he dies . . . he can do anything he wants to do—there is no concept of marital rape” [Asian migrant victim living in New Zealand] (Tse, 2007, p. 176). Women were taught by their community to accept and tolerate violence within a marriage: “. . . the way we were brought up, you know in your house they will tell you, in your husband’s house, this is what will happen, you don’t have to complain” [Nigerian victim living in England] (Femi-Ajao., 2018, p. 5).

Marriage was described by participants as a sacrament and divorce was considered taboo, contributing to pressure for women to maintain a harmonious relationship. Failure to maintain family harmony was perceived as the woman’s fault and a justification for violence: “. . . If I change myself, listen to my husband, tolerate him, this will bring happiness to my family” [South Asian migrant victim living in Canada] (Ahmad et al., 2009, p. 618). Even in cultures where divorce was acceptable, there was stigma and shame attributed to divorced women and single-parent families. Women seeking formal support were often perceived as going against marital obligations and in doing so, damaging community reputation. Fear of shame and ostracization prevented women from seeking support, particularly from formal sources, whose intentions were often perceived as being at odds with family harmony.

##### Self-sacrificing women

Twenty-six studies (see Appendix D) identified the expectation for women to maintain family harmony and to tolerate the abuse for others’ sake. While Hispanic cultures describe this expectation as “aguarantos” (Falconier et al., 2013), similar expectations were described by many cultural groups. Many women viewed their tolerance and resilience as part of their cultural identity; African American women described themselves as “strong black women” (Monterrosa, 2019, p. 9), and “strong-minded” (Sears, 2021); Jamaican women described themselves as being “hammer-strong” (Sears, 2021); Nigerian women viewed themselves as belonging to a culture of “suffering and smiling” (Femi-Ajao, 2018, p. 5); and Chuukese women saw enduring suffering as a mark of character (Magnussen et al., 2011). The ability and willingness to tolerate the abuse was seen as a sign of strength that encouraged women to be self-reliant and not depend on the support of others to manage the violence, deterring the need for formal support.

Although it was the expectation of many communities for women to tolerate the abuse, many victim-survivors described a willingness to tolerate the abuse for the sake of their children. Tolerating abuse was described as “choosing the devil they knew over the devil they did not” [Latina migrant victim living in North America] (Kelly, 2009, p. 9), indicating that women believed that staying in the relationship was a safer option for their children. Perpetrators often exploited women’s distrust and fear of services, particularly for immigrant women, as a means of discouraging women from disclosing the abuse: “he said that I was going to be deported, that INS [immigration and naturalization service] would send me to Mexico and they would take my children” [migrant victim] (Erez & Globokar, 2009, p. 134). The safety and well-being of children were pivotal in women’s decisions to withstand the abuse. Concerns for their children’s welfare often outweighed the influence of norms that prevented them from seeking formal help.

#### Theme 2: Community recognition and acceptance of abuse

This theme describes how the community has a significant influence in shaping women’s perceptions and responses to IPV. The subsequent subthemes describe how the normalization and acceptance of abuse among the community can impact women’s recognition of the need to seek formal help.

##### Normalization of abuse

Participants across 21 studies (see Appendix D) discussed how at least some level of violence between intimate partners was considered normal and commonplace within their community: “. . . I didn’t know that was abuse . . . I thought that was normal” [Latina migrant victim living in Iowa] (Reina et al., 2014, p. 602). Intergenerational violence was often described as contributing to their acceptance of IPV: “you see the parents fighting and the father beating up the mother and the kids grow up thinking that’s okay” [service provider for Latina Community in New Jersey] (Lewis et al., 2005, p. 76).

The normalization of violence by the community resulted in unresponsiveness which was described as the “community’s blind eye” [migrant victim living in Canada] (Ahmad et al., 2009, p. 617). Even where women had sought help from their community, responses had reinforced the normalization of IPV and encouraged women to stay silent: “yes, it’s normal . . . If you tell (anyone), your peers will ask you, ‘is this your first time to be beaten?’” [female Tanzanian community member] (McCleary-Sills et al., 2016, p. 230). Women felt that formal intervention was not warranted and instead relied on peer support, taking comfort from sharing experiences with those in the same situation: “other wives I know said the same thing, ‘oh yes, my husband hits me too; that’s normal’, so that is how we believed, and how we all survived” [African migrant victim living in the United States] (Ting, 2010, p. 353).

##### Community acceptance moderated by severity

The acceptance of IPV was however moderated by severity, particularly where children were at risk, as described in seven studies (see Appendix D). Participants in the McCleary-Sills et al. (2016) study described that severe physical abuse, public physical abuse, and anal rape against a partner were not tolerated by the community and warranted formal intervention. Similarly, several participants discussed that where the violence escalated, either they individually recognized, or their informal supports recognized the need for formal assistance: “in our culture, they say that they have to talk to closest friends and leaders to help them out with these things, but if it is worse, then they can go to other services” [ethnic minority woman living in the United States] (Wolf et al., 2003, p. 124). Such responses highlight the pivotal role that the community has in influencing women’s perception of violence and their willingness to seek formal support.

#### Theme 3: Honor-based society

This theme describes how honor-based cultures tend to value the collective and the reputation of their community over the needs of the individual. The subsequent section describes how honor and shame can discourage women from seeking assistance, particularly from supports that are considered outside of the community.

##### Defending the collective

Twenty-eight studies (specified in Appendix D) discussed the concept of shame in relation to disclosing abuse. These studies described cultural values that align with the principles of collectivism, though various terms were used including familism, familialism, familsmo, family solidarity, loyalty to community, or being family-oriented. These values centered around participants viewing themselves as an extension of their family, clan, or community and the expectation and willingness to prioritize the needs of the collective above the needs of the individual at all costs (Magnussen et al., 2011).

Honor was described in reference to saving face, protecting the family name, and protecting community reputation. Disclosing the abuse was considered an act of dishonor, often referred to as “airing dirty laundry” (Tam et al., 2016, p. 233). Disclosing the abuse was described as bringing shame on the individual and the community and discouraging help-seeking: “I don’t want to tell anybody what’s happening. And I feel ashamed to tell anybody what’s going on in the house” [Asian migrant victim living in the United States] (Bauer et al., 2000, p. 38). This sense of shame seemed to be heightened when disclosing the abuse to those outside the community and warranted an ostracization of the individual or, as described by a South-Korean woman in the study by Park and Ko (2021), self-isolation of the victim-survivor, “. . . if I report to the police, once such a thing is open to the public, I would be so disgraced that I may not be able to show my face any longer when I go out” (p. 331).

##### Collective as protective

Though collectivist values can incite shame in women disclosing violence, six studies (see Appendix D) described how collectivist cultures also supported women by offering protection, emotional, practical support, and perpetrator accountability. As described by a migrant victim in Ahmad et al. (2009), women who immigrate from collectivist cultures to more individualist cultures experience a sense of isolation: “back home if something like this happens, all village people go with sticks to tell the man that it should not happen, but here, we do not have anyone” (p. 1). Though the support of the community at times reduced the perceived need for formal support, many women described how community support assisted them with identifying the abuse and provided opportunities to learn about and access formal services.

#### Theme 4: Role of religion

Seventeen studies (see Appendix D) discussed the role of religion and religious institutes in shaping cultural norms and their influence on willingness to seek help. The subthemes below elaborate on how women’s reliance on religious beliefs and leaders for support and strength can further normalize violence and discourage formal help-seeking.

##### Religious beliefs as a coping mechanism

Many women described utilizing prayer as a means of seeking help to keep themselves safe and providing them with the strength to continue to endure the abuse: “with continuous prayers, I could gain strength from God . . .” [Asian migrant victim living in New Zealand] (Tse, 2007, p. 187). Many women described turning to God to seek solutions and described God as giving them hope that the suffering would end and justice would be served: “I believe that God will bring justice one day to this man for what he has done to me . . .” [African migrant victim living in the United States] (Ting & Panchanadeswaran, 2009, p. 43). Similarly, some women described the belief that God had a reason they were being made to suffer: “I believe that God will take care for me, that God has a reason for having me suffer” [African migrant victim living in the United States] (Ting, 2010, p. 352). Such beliefs provide women with a sense of hope and reduce the need for women to seek help from formal services.

##### Influence of religious leaders

Religious leaders were reported to provide emotional support, as described by a Mexican migrant victim-survivor in Acevedo (2000) “at least I felt listened to. I was able to vent. I wasn’t as worried afterwards. It was like therapy” (p. 269). In some instances, religious leaders acted as a mediators, or as figures of authority, assisting in holding the perpetrator to account and actively supporting women to leave abusive relationships. However, as described by a Vietnamese migrant victim in Bui and Morash’s (2007) study, they were also found to reinforce norms that silence women and promote family unity as being above the woman’s well-being: “when I went to the Buddhist temple, I listened to the advice of the monk, and I tried to tolerate [my husband’s] behavior and forgive him” (p. 384). This reliance and trust in religious leaders deterred women from seeking help from other formal services.

#### Theme 5: Cultural beliefs and attitudes toward formal services

The four previous themes contribute to the individual and group cultural beliefs and attitudes toward formal services. The subthemes below describe the specific cultural beliefs and attitudes toward formal services that can actively discourage women’s willingness to engage with formal services.

##### IPV must be physical or severe

Fourteen studies (see Appendix D) discussed the belief that IPV must be either physical or extremely severe to warrant the intervention of formal services, “I did not have any physical injuries . . . So, I hesitated to call the police. I thought they might scold me for reporting something trivial” [South Korean victim living in South Korea] (Park & Ko, 2021, p. 331). In some instances, these beliefs were a result of previous experiences with formal services, as described in a study by Mookerjee et al. (2015): “it (experience of getting an order of protection) was not good because they did not accept it because it was not physical abuse” [Hispanic victim] (p. 843). The perception that IPV must be severe often deterred or delayed women from accessing supports until the violence had escalated beyond a tolerance level that they themselves or their informal supports could cope with.

##### Formal services are a last resort

Participants described a hierarchy in help-seeking with most women choosing to first seek the support of informal supports and as described above, accessing formal supports only when informal supports were insufficient. This hierarchy is influenced by beliefs that formal services should only be used for very severe IPV and intergenerational expectations that women should be self-reliant and try to “take matters into their own hands” [ethnic minority victim] (Wolf et al., 2003, p. 183). Intergenerational messages of service avoidance were often a result of previous experiences of racist and discriminatory practices. As described in Nicolaidis et al. (2010), older generations had been denied access to formal health care and as a result, had taught their children not to trust formal services. The reluctance to seek support often led to women engaging with services at the point of crisis rather than prevention or intervention as described by a female Latina community member in Falconier et al. (2013): “the majority of Hispanics don’t seek help . . . the majority of Hispanics don’t seek help until the problem is too advanced” (p. 363). The role of informal support in encouraging early access to formal services may be critical with some participants describing how informal supports provided them with the knowledge and support to engage with other formal services, creating a snowball effect of engagement.

##### Victims fear consequences of service engagement

Twenty-three studies (see Appendix D) discussed victim-survivors’ fears about engaging with formal services including the fear of threatened family solidarity, ostracization by the community, escalating violence, family retribution, deportation, and the removal of children by formal services. The fear that intervention could threaten family solidarity was particularly common and was attributed to the shame that would be brought on the woman, the family, and even the broader community if formal services were involved. Formal services were often described as disrespectful of cultural values of family and were perceived as having a primary goal of removing women from the relationship, which was in direct conflict with the community wishes to maintain family unity (Bhuyan et al., 2005).

##### Women do not have equal rights or access to services

Gender norms and community acceptance of IPV often led to perceptions that women did not have equal rights to men. For some women living in areas such as Africa, these perceptions were founded: IPV and marital rape were not considered illegal. Migrant victim-survivors in the Ting (2010) study described: “in Africa, you can go to the police, and they will laugh at you and send you home. They tell you your husband is right to discipline you” (p. 355). However, even where IPV is penalized and women have equal rights to men, women still believed that they did not have the right or ability to access services. This may be a consequence of poor awareness of their rights and the availability of services or a result of previous experiences with formal responses to IPV. Immigrant women were particularly influenced by these perceptions due to greater barriers in accessing the required information about laws, rights, and services available to them. As described by a victim-survivor in Shen (2010) “I was helpless. I didn’t know whom I could ask for help. I didn’t know whom I could talk with” (p.14).

##### Service responses penalize women

Women across 24 studies (see Appendix D) described a range of perceptions and experiences about the ineffectiveness and inadequacy of service responses to women. Descriptions of ineffective responses included: delayed responses, inaction toward perpetrators, blaming women for the abuse, an absence of services to respond to IPV issues, and a lack of inquiry into IPV, with women describing “. . . I didn’t tell him about the violence because he didn’t ask” [Latina migrant victim living in California] (Rodriguez et al., 1998, p. 310). Previous experiences of ineffective services impacted women’s future service engagement, with women stating that “I didn’t really think of shelters, because I called once and they never came for me” [Mexican migrant victim] (Acevedo, 2000, p. 272). Similarly, women, expressed distrust in services, “It (restraining order) means absolutely nothing to my ex-partner and to the police” [ethnic minority victim] (Tam et al., 2015, p. 532). Such experiences were re-traumatizing for victims and deterred them from seeking formal assistance.

##### Services are not culturally relevant

The ineffectiveness, inadequacy, and cultural irrelevance of services were described in 24 studies (see Appendix D). As described earlier, the belief that services don’t respect the cultural values of family and collectivism was described in eight studies and was perceived as threatening to family solidarity. Women expressed the desire for services to work with the family to stay together: “I think that if a family comes to a professional person to be listened to and be given advice, he/she can calm her down and then call the husband, and should not disintegrate that” [Latino health promotor] (Falconier et al., 2013, p. 371). However, women described being asked to conform to mainstream approaches, such as reporting violence, or risk having the support of services withdrawn (Guruge & Humphreys, 2009). An ethnic minority victim-survivor in Tam et al. (2016), also described how the misalignment of their views with the service providers discouraged service engagement:

This lawyer does not understand my culture. Sometimes, I explained to the lawyer my views, but he was unable to understand why I have such views or why I made such decision (to give up her child's custody to her husband). (p. 534)

Other practical barriers that reduced the cultural relevance of services included the lack of culturally diverse workers and the lack of services that were linguistically accessible. Women reported that inquiry by practitioners helps to initiate conversations about IPV and reduce the fear of shame from the service provider: “. . . I say if the doctors would have insisted or told me more things than what they told me, but because they stopped insisting, I did not say the truth” [ethnic minority victim living in the United States] (Gonzalez-Guarda et al., 2016, p. 234).

## Discussion

This meta-synthesis integrated findings gathered from a systematic search of qualitative studies on the influence of cultural norms on service engagement. Based on thematic synthesis, the following five cultural norms were themes that describe factors that may explain why non-Anglo-Saxon victims of IPV choose not to seek formal help: (a) gender roles and social expectations, (b) community recognition and acceptance of abuse, (c) honor-based society, (d) the role of religion, and (e) cultural beliefs and attitudes toward formal services. In what follows, the identified themes are discussed in relation to prior research and theory.

The meta-synthesis identified that cultural norms that prescribe how women should behave had a significant influence on women’s ability to recognize the abuse as problematic, impacting the first stage of help-seeking (Liang et al., 2005). Intergenerational cultural norms that view women with little decision-making power and as subservient to men (e.g., Femi-Ajao, 2018; Lewis et al., 2005; Tse, 2007), in addition to exposure to IPV in their upbringings, resulted in a belief that violence is commonplace (Lewis et al., 2005). These beliefs were further reinforced by community and societal responses to violence. Women reported being encouraged by friends and family to placate and obey their partners to avoid conflict (e.g., Ahmad et al., 2009; McCleary-Sills et al., 2016) and in other circumstances, the violence was completely ignored by the community, referred to by some as “turning a blind eye” (Ahmad et al., 2009). These casual attitudes from friends and family around female subservience and violence against women further normalized the abuse and discouraged further help-seeking (e.g., Acevedo, 2000; Lewis et al., 2005). Cross-cultural studies of IPV have indicated that IPV occurs more frequently in societies where conflict is considered commonplace, and ideologies of male dominance prevail (Levinson, 1988). The unequal positioning of women within intimate relationships and within society coupled with the normative use of violence to resolve conflict, serve to legitimize the use of violence against women as discipline (Jewkes, 2002).

Unequal positioning of women can often be reinforced by religious institutions (Hunnicutt, 2009). For example, in this meta-synthesis, the impact of religious institutions was often two-fold: (a) women described religious norms and practices that prioritized the unity of marriage above the safety and well-being of women, and (b) while religious leaders provided support to women, they either advertently or inadvertently reinforced that women should accept and tolerate the abuse, rely on prayer and internal coping mechanisms rather than formal services for support (e.g., Bui & Morash, 2007; Shen, 2011).

Notions of shame and dishonor also contribute to women’s decision to remain in abusive relationships. Women explained that disclosing abuse, particularly to those outside of the community was a dishonorable act that did not conform to ideas of being a “good wife” and would bring shame on the family and the broader community (e.g., Magnussen et al., 2011; Tam et al., 2015). For many of these women, seeking a separation would result in an ostracization from the community. In addition to attributed shame and dishonor associated with separation, as described by Jewkes (2002), in communities where women are considered to be of lower status, they often have limited social and economic means to leave a relationship and, in some instances, limited legal access or laws that will support them. As such, women are further deterred from seeking help.

Consistent with the help-seeking theory proposed by Liang et al. (2005), individuals’ definitions of violence were shaped by the social context within which they are situated. As identified in the first four themes, women’s perceived experience of abuse was influenced by the context of gender roles, community responses to violence, shame and honor and the influence of other macrosocial factors such as religious norms (Liang et al., 2005). The norms described in the four previous themes contribute to the beliefs and attitudes toward formal service engagement as described in the fifth theme.

Women described formal services to be ineffective, penalizing or blaming women; discriminatory and racist; insensitive to cultural values, and lacking in cultural resources (e.g., Bauer et al., 2000; Keller & Brennan, 2007; Park & Ko, 2021). Women described an intergenerational distrust in formal services that had resulted in women believing they had only themselves to rely on (e.g., Nicolaidis et al., 2010; Wolf et al., 2003; Li et al., 2022). Services were perceived as outsiders and a threat to the community due to goals that sought to separate families, rather than unify them (e.g., Kaukinen & DeMaris, 2009; Kim & Hogge, 2015). For some women, the distrust in services was a result of previous experience with the same services. For many women, the countries in which they were living had inadequate laws to protect women from violence (Park & Ko, 2021). For other women, particularly migrant and ethnic minority women, their host country had adequate laws to protect them from violence; however, the service responses were discriminatory, ineffective, or not culturally responsive (e.g., Shen, 2011; Tam et al., 2016). The reluctance to engage with formal services as identified in this meta-synthesis, has been reflected in previous studies which have found that prior unsuccessful engagement with services and perceived cultural insensitivity discourage women from non-Anglo-Saxon communities accessing formal supports (Fraser et al., 2002). Previous help-seeking literature has shown that women from non-Anglo- Saxon communities experience unique barriers to service engagement such as racial discrimination and unequal treatment when seeking support, including higher rates of child removal (e.g., Dosanjh et al., 2008; Landrine et al., 2006; Smedley et al., 2003). Such distrust in services and the reliance on self-coping or informal supports is consistent with help-seeking literature that suggests women experiencing IPV may progress from private attempts to manage the abuse to more public help-seeking only as the situation worsens (Abraham, 2000; Goodman et al., 2003) and may explain why non-Anglo-Saxon communities often only access formal services at the point of crisis (Ergöçmen et al., 2013; Satyen et al., 2018).

The findings of this meta-synthesis indicate several cultural norms that influence non-Anglo-Saxon women’s engagement with formal services. Though many of the factors identified in the meta-synthesis are not unique to non-Anglo-Saxon communities, their experience of gender inequality, normalized abuse by the community and religious leaders and associated shame of experiencing IPV are more accentuated when considering these factors as intersecting and compounding. Though there are limited evaluations of initiatives that target cultural norms specifically, the Communities Care Program, led by the United Nations Children’s fund, found that challenging norms around sexual violence, protecting family honor, and husband’s right to use violence led to a change in the community’s tolerance of violence and consequently, the effective engagement of women in formal services (Glass et al., 2018). Altering service responses to women experiencing violence is an additional approach to improving formal service engagement of non-Anglo-Saxon women. Harrison et al. (2019) identified building foundations of trust and respect, diversifying communication channels, generating community partnerships, and taking the time to understand the needs of non-Anglo-Saxon communities as essential to successful service engagement. Service responses to non-Anglo-Saxon women need to consider the cultural context of the individual and the influence of cultural norms to ensure that service responses are better tailored to their needs.

### Strengths and Limitations

This meta-synthesis utilized a comprehensive and systematic search strategy to capture the relevant studies for inclusion with participants from several countries and cultures, enabling a thorough understanding of similarities and differences across non-Anglo-Saxon groups. Similarly, the focus of examining only cultural norms on formal help-seeking allowed for a more thorough analysis of how culture may influence help-seeking, depending on the type of support available. The findings highlight the need for formal service providers to better meet the needs of non-Anglo-Saxon communities and build trust to enable greater service engagement.

There are limitations to this review that should be considered when interpreting the findings. This meta-synthesis only included studies that were written in English. Given that this study is observing non-Anglo-Saxon communities, other-language research relevant to this topic may have been omitted. This meta-synthesis identified immigrant and non-immigrant populations but did not analyze the findings as two distinct groups. As such, there may be unique challenges between these two groups that have not been identified in this study. Despite the limitations, the findings contribute to a greater understanding of the influence of cultural norms on formal help-seeking.

## Conclusions

This is the first qualitative meta-synthesis that considers the influence of cultural norms on formal help-seeking behavior. The meta-synthesis has captured the experiences of non-Anglo-Saxon women and their experience of cultural norms and beliefs that prevent them from accessing formal services in a manner that quantitative data cannot. By synthesizing the qualitative insights of participants, we were able to draw comparisons and similarities across cultural groups and identify cultural norms that commonly discourage formal help-seeking behavior. These findings provide context to the existing quantitative literature that demonstrates the varied nature in which non-Anglo-Saxon communities engage with formal services. The family violence sector and other service providers who seek to engage non-Anglo-Saxon communities can benefit from this meta-synthesis by considering how their services can be more culturally responsive to the needs of diverse communities. Given the vulnerability of women in diverse communities and the context of increasing multiculturalism, greater focus is required on reducing IPV in this population while paying respect to their cultural values.

## Supplemental Material

sj-docx-1-tva-10.1177_15248380231162971 – Supplemental material for Influence of Cultural Norms on Formal Service Engagement Among Survivors of Intimate Partner Violence: A Qualitative Meta-synthesis Click here for additional data file. Supplemental material, sj-docx-1-tva-10.1177_15248380231162971 for Influence of Cultural Norms on Formal Service Engagement Among Survivors of Intimate Partner Violence: A Qualitative Meta-synthesis by Jane Green, Lata Satyen and John W. Toumbourou in Trauma, Violence, & Abuse

sj-docx-2-tva-10.1177_15248380231162971 – Supplemental material for Influence of Cultural Norms on Formal Service Engagement Among Survivors of Intimate Partner Violence: A Qualitative Meta-synthesis Click here for additional data file. Supplemental material, sj-docx-2-tva-10.1177_15248380231162971 for Influence of Cultural Norms on Formal Service Engagement Among Survivors of Intimate Partner Violence: A Qualitative Meta-synthesis by Jane Green, Lata Satyen and John W. Toumbourou in Trauma, Violence, & Abuse

sj-docx-3-tva-10.1177_15248380231162971 – Supplemental material for Influence of Cultural Norms on Formal Service Engagement Among Survivors of Intimate Partner Violence: A Qualitative Meta-synthesis Click here for additional data file. Supplemental material, sj-docx-3-tva-10.1177_15248380231162971 for Influence of Cultural Norms on Formal Service Engagement Among Survivors of Intimate Partner Violence: A Qualitative Meta-synthesis by Jane Green, Lata Satyen and John W. Toumbourou in Trauma, Violence, & Abuse

sj-docx-4-tva-10.1177_15248380231162971 – Supplemental material for Influence of Cultural Norms on Formal Service Engagement Among Survivors of Intimate Partner Violence: A Qualitative Meta-synthesis Click here for additional data file. Supplemental material, sj-docx-4-tva-10.1177_15248380231162971 for Influence of Cultural Norms on Formal Service Engagement Among Survivors of Intimate Partner Violence: A Qualitative Meta-synthesis by Jane Green, Lata Satyen and John W. Toumbourou in Trauma, Violence, & Abuse
