# Supplementary material for: Influence of Cultural Norms on Formal Service Engagement Among Survivors of Intimate Partner Violence: A Qualitative Meta-synthesis
Source: Trauma Violence Abuse. 2023 Apr 19;25(1):738–51. doi: 10.1177/15248380231162971 (PMC10666477; doi:10.1177/15248380231162971)
Supplement: sj-docx-1-tva-10.1177_15248380231162971 – Supplemental material for Influence of Cultural Norms on Formal Service Engagement Among Survivors of Intimate Partner Violence: A Qualitative Meta-synthesis [file sj-docx-1-tva-10.1177_15248380231162971.docx]

**Appendix A**

*Comprehensive Search Strategy*

**Embase**

| #1 | norm*:ab,ti |
| --- | --- |
| #2 | stigma:ab,ti |
| #3 | attitud*:ab,ti |
| #4 | stereotyp*:ab,ti |
| #5 | prejudic*:ab,ti |
| #6 | belief*:ab,ti |
| #7 | 'tradition':ab,ti |
| #8 | sociocultur*:ab,ti |
| #9 | custom*:ab,ti |
| #10 | value*:ab,ti |
| #11 | factor*:ab,ti |
| #12 | trait*:ab,ti |
| #13 | 'cultur* differen*':ab,ti |
| #14 | 'ethnic* differen*':ab,ti |
| #15 | 'cultural value'/de |
| #16 | 'social norm'/de |
| #17 | 'help seek*':ab,ti |
| #18 | 'help-seek*':ab,ti |
| #19 | 'assistance seek*':ab,ti |
| #20 | 'service access':ab,ti |
| #21 | 'service use':ab,ti |
| #22 | utili?ation:ab,ti |
| #23 | utili?e:ab,ti |
| #24 | engag*:ab,ti |
| #25 | 'information seek*':ab,ti |
| #26 | barrier*:ab,ti |
| #27 | 'help seeking'/de |
| #28 | 'partner violence':ab,ti |
| #29 | 'partner abuse':ab,ti |
| #30 | 'relationship violence':ab,ti |
| #31 | 'couple violence':ab,ti |
| #32 | 'dating violence':ab,ti |
| #33 | 'domestic violence':ab,ti |
| #34 | 'family violence':ab,ti |
| #35 | 'domestic abuse':ab,ti |
| #36 | 'ipv':ab,ti |
| #37 | 'marital violence':ab,ti |
| #38 | 'battered wom?n':ab,ti |
| #39 | 'battered female':ab,ti |
| #40 | 'spous* violence':ab,ti |
| #41 | 'spous* abuse':ab,ti |
| #42 | 'wife abuse':ab,ti |
| #43 | 'wife batter*':ab,ti |
| #44 | 'wife beat*':ab,ti |
| #45 | 'domestic violence'/exp |
| #46 | ethnic*:ab,ti |
| #47 | race:ab,ti |
| #48 | racial:ab,ti |
| #49 | migrant*:ab,ti |
| #50 | immigrant*:ab,ti |
| #51 | refugee*:ab,ti |
| #52 | 'english as a second language*':ab,ti |
| #53 | esl:ab,ti |
| #54 | foreign*:ab,ti |
| #55 | 'ethnic minorit*':ab,ti |
| #56 | divers*:ab,ti |
| #57 | asylum:ab,ti |
| #58 | 'cultur* group':ab,ti |
| #59 | 'multicultur* group':ab,ti |
| #60 | 'ethnic group'/de |
| #61 | #1 OR #2 OR #3 OR #4 OR #5 OR #6 OR #7 OR #8 OR #9 OR #10 OR #11 OR #12 OR #13 OR #14 OR #44 OR #45 |
| #62 | #15 OR #16 OR #17 OR #18 OR #19 OR #20 OR #21 OR #22 OR #23 OR #24 OR #25 |
| #63 | #26 OR #27 OR #28 OR #29 OR #30 OR #31 OR #32 OR #33 OR #34 OR #35 OR #36 OR #37 OR #38 OR #39 OR #40 OR #41 OR #42 OR #43 |
| #64 | #46 OR #47 OR #48 OR #49 OR #50 OR #51 OR #52 OR #53 OR #54 OR #55 OR #56 OR #57 OR #58 OR #59 OR #60 |
| #65 | #61 AND #62 AND #63 AND #64 |
| #66 | #61 AND #62 AND #63 AND #64 AND ([adult]/lim OR [young adult]/lim OR [middle aged]/lim OR [aged]/lim OR [very elderly]/lim) AND [humans]/lim AND [english]/lim AND [embase]/lim |
| **Limiters** | Humans, English, Adulthood, Embase. |
| **Definitions** | :ti – title, :ab – abstract, :de – exact subject heading, :exp.- exploded subject heading |

**Medline (via Embase)**

| #1 | norm*:ab,ti |
| --- | --- |
| #2 | stigma:ab,ti |
| #3 | attitud*:ab,ti |
| #4 | stereotyp*:ab,ti |
| #5 | prejudic*:ab,ti |
| #6 | belief*:ab,ti |
| #7 | 'tradition':ab,ti |
| #8 | sociocultur*:ab,ti |
| #9 | custom*:ab,ti |
| #10 | value*:ab,ti |
| #11 | factor*:ab,ti |
| #12 | trait*:ab,ti |
| #13 | 'cultur* differen*':ab,ti |
| #14 | 'ethnic* differen*':ab,ti |
| #15 | 'cultural value'/de |
| #16 | 'social norm'/de |
| #17 | 'help seek*':ab,ti |
| #18 | 'help-seek*':ab,ti |
| #19 | 'assistance seek*':ab,ti |
| #20 | 'service access':ab,ti |
| #21 | 'service use':ab,ti |
| #22 | utili?ation:ab,ti |
| #23 | utili?e:ab,ti |
| #24 | engag*:ab,ti |
| #25 | 'information seek*':ab,ti |
| #26 | barrier*:ab,ti |
| #27 | 'help seeking'/de |
| #28 | 'partner violence':ab,ti |
| #29 | 'partner abuse':ab,ti |
| #30 | 'relationship violence':ab,ti |
| #31 | 'couple violence':ab,ti |
| #32 | 'dating violence':ab,ti |
| #33 | 'domestic violence':ab,ti |
| #34 | 'family violence':ab,ti |
| #35 | 'domestic abuse':ab,ti |
| #36 | 'ipv':ab,ti |
| #37 | 'marital violence':ab,ti |
| #38 | 'battered wom?n':ab,ti |
| #39 | 'battered female':ab,ti |
| #40 | 'spous* violence':ab,ti |
| #41 | 'spous* abuse':ab,ti |
| #42 | 'wife abuse':ab,ti |
| #43 | 'wife batter*':ab,ti |
| #44 | 'wife beat*':ab,ti |
| #45 | 'domestic violence'/exp |
| #46 | ethnic*:ab,ti |
| #47 | race:ab,ti |
| #48 | racial:ab,ti |
| #49 | migrant*:ab,ti |
| #50 | immigrant*:ab,ti |
| #51 | refugee*:ab,ti |
| #52 | 'english as a second language*':ab,ti |
| #53 | esl:ab,ti |
| #54 | foreign*:ab,ti |
| #55 | 'ethnic minorit*':ab,ti |
| #56 | divers*:ab,ti |
| #57 | asylum:ab,ti |
| #58 | 'cultur* group':ab,ti |
| #59 | 'multicultur* group':ab,ti |
| #60 | 'ethnic group'/de |
| #61 | #1 OR #2 OR #3 OR #4 OR #5 OR #6 OR #7 OR #8 OR #9 OR #10 OR #11 OR #12 OR #13 OR #14 OR #44 OR #45 |
| #62 | #15 OR #16 OR #17 OR #18 OR #19 OR #20 OR #21 OR #22 OR #23 OR #24 OR #25 |
| #63 | #26 OR #27 OR #28 OR #29 OR #30 OR #31 OR #32 OR #33 OR #34 OR #35 OR #36 OR #37 OR #38 OR #39 OR #40 OR #41 OR #42 OR #43 |
| #64 | #46 OR #47 OR #48 OR #49 OR #50 OR #51 OR #52 OR #53 OR #54 OR #55 OR #56 OR #57 OR #58 OR #59 OR #60 |
| #65 | #61 AND #62 AND #63 AND #64 |
| #66 | #61 AND #62 AND #63 AND #64 AND ([adult]/lim OR [young adult]/lim OR [middle aged]/lim OR [aged]/lim OR [very elderly]/lim) AND [humans]/lim AND [english]/lim AND [medline]/lim |
| **Limiters** | Humans, English, Adulthood, Medline. |
| **Definitions** | :ti – title, :ab – abstract, :de – exact subject heading, :exp.- exploded subject heading |

**ERIC (via EBSCOhost)**

| S1 | TI norm* or AB norm* |
| --- | --- |
| S2 | TI stigma or AB stigma |
| S3 | TI attitud* or AB attitud* |
| S4 | TI belief* or AB belief* |
| S5 | TI tradition* or AB tradition* |
| S6 | TI custom* or AB custom* |
| S7 | TI value* or AB value* |
| S8 | TI trait* or AB trait* |
| S9 | TI stereotyp* or AB stereotyp* |
| S10 | TI prejudic* or AB prejudic* |
| S11 | TI factor* or AB factor* |
| S12 | TI "cultur* differen*" or AB "cultur* differen*" |
| S13 | TI "ethnic* differen*" or AB "ethnic* differen*" |
| S14 | DE "Cultural Traits" |
| S15 | DE "Cultural Influences" |
| S16 | DE "Social Values" |
| S17 | S1 OR S2 OR S3 OR S4 OR S5 OR S6 OR S7 OR S8 OR S9 OR S10 OR S11 OR S12 OR S13 OR S14 OR S15 OR S16 |
| S18 | TI help-seek* or AB help-seek* |
| S19 | TI "help seek*" or AB "help seek*" |
| S20 | TI "assistance seek*" or AB "assistance seek*" |
| S21 | TI "service access" or AB "Service access" |
| S22 | TI "service use" or AB "service use" |
| S23 | TI "utili?ation N2 service*" or AB "utili?ation N2 service*" |
| S24 | TI "utili? N2 service*" or AB "utili? N2 service*" |
| S25 | TI “engag* N2 service*” or AB “engag* N2 service*” |
| S26 | TI "information seek*" or AB "information seek*" |
| S27 | TI barrier* or AB barrier* |
| S28 | DE "Help seeking" |
| S29 | S18 OR S19 OR S20 OR S21 OR S22 OR S23 OR S24 OR S25 OR S26 OR S27 OR S28 |
| S30 | TI “partner violence” OR AB “partner violence” |
| S31 | TI “partner abuse” OR AB “partner abuse” |
| S32 | TI “relationship violence” OR AB “relationship violence” |
| S33 | TI “couple violence” OR AB “couple violence” |
| S34 | TI “dating violence” OR AB “dating violence” |
| S35 | TI “domestic violence” OR AB “domestic violence” |
| S36 | TI “family violence” OR AB “family violence” |
| S37 | TI “domestic abuse” OR AB “domestic abuse” |
| S38 | TI IPV OR AB IPV |
| S39 | TI “marital violence” OR AB “marital violence” |
| S40 | TI “battered wom?n” OR AB “battered wom?n” |
| S41 | TI “battered female*” OR AB “battered female*” |
| S42 | TI “spous* violence” OR AB “spous* violence” |
| S43 | TI “spous* abuse” OR AB “spous* abuse” |
| S44 | TI “wife abuse” OR AB “wife abuse” |
| S45 | TI “wife beat*” OR AB “wife beat*” |
| S46 | TI "wife batter*" OR AB "wife batter*" |
| S47 | DE "Family Violence" |
| S48 | S30 OR S31 OR S32 OR S33 OR S34 OR S35 OR S36 OR S37 OR S38 OR S39 OR S40 OR S41 OR S42 OR S43 OR S44 OR S45 OR S46 OR S47 |
| S49 | TI "ethnic minorit*"" or AB "ethnic minorit*" |
| S50 | TI ethnic* OR AB ethnic* |
| S51 | TI race OR AB race |
| S52 | TI racial OR AB racial |
| S53 | TI migrant* OR AB migrant* |
| S54 | TI immigrant* OR AB immigrant* |
| S55 | TI refugee* OR AB refugee* |
| S56 | TI “English as a second language” OR AB “English as a second language” |
| S57 | TI ESL OR AB ESL |
| S58 | TI foreign* OR AB foreign* |
| S59 | TI minorit* OR AB minorit* |
| S60 | TI divers* OR AB divers* |
| S61 | TI asylum or AB asylum |
| S62 | TI "mulitcultur* group" or AB "multicultur* group" |
| S63 | TI "cultur* group" or AB "cultur* group" |
| S64 | DE "Ethnic Groups" OR DE "Alaska Natives" OR DE "American Indians" OR DE "Anglo Americans" OR DE "Canada Natives" OR DE "Chinese Americans" OR DE "Eskimos" OR DE "Filipino Americans" OR DE "French Canadians" OR DE "Hawaiians" OR DE "Hmong People" OR DE "Italian Americans" OR DE "Japanese Americans" OR DE "Korean Americans" OR DE "Mexican Americans" OR DE "Pacific Islanders" OR DE "Polish Americans" OR DE "Samoan Americans" OR DE "Spanish Americans" |
| S65 | S49 OR S50 OR S51 OR S52 OR S53 OR S54 OR S55 OR S56 OR S57 OR S58 OR S59 OR S60 OR S61 OR S62 OR S63 OR S64 |
| S66 | S17 AND S29 AND S48 AND S65 |
| **Limiters** | Peer-reviewed, English |
| **Definitions** | Ti – title, Ab – abstract, DE – exact subject heading |

**APA PsycInfo**

| S1 | TI norm* or AB norm* |
| --- | --- |
| S2 | TI stigma or AB stigma |
| S3 | TI attitud* or AB attitud* |
| S4 | TI belief* or AB belief* |
| S5 | TI tradition* or AB tradition* |
| S6 | TI custom* or AB custom* |
| S7 | TI value* or AB value* |
| S8 | TI trait* or AB trait* |
| S9 | TI stereotyp* or AB stereotyp* |
| S10 | TI prejudic* or AB prejudic* |
| S11 | TI factor* or AB factor* |
| S12 | TI "cultur* differen*" or AB "cultur* differen*" |
| S13 | TI "ethnic* differen*" or AB "ethnic* differen*" |
| S14 | DE Ethnic Values |
| S15 | DE Social Values |
| S16 | DE Social Norms |
| S17 | S1 OR S2 OR S3 OR S4 OR S5 OR S6 OR S7 OR S8 OR S9 OR S10 OR S11 OR S12 OR S13 OR S14 OR S15 OR S16 |
| S18 | TI help-seek* or AB help-seek* |
| S19 | TI "help seek*" or AB "help seek*" |
| S20 | TI "assistance seek*" or AB "assistance seek*" |
| S21 | TI "service access" or AB "Service access" |
| S22 | TI "service use" or AB "service use" |
| S23 | TI "utili?ation N2 service*" or AB "utili?ation N2 service*" |
| S24 | TI "utili? N2 service*" or AB "utili? N2 service*" |
| S25 | TI "engag* N2 service*" or AB "engag* N2 service*" |
| S26 | TI "information seek*" or AB "information seek*" |
| S27 | TI barrier* or AB barrier* |
| S28 | DE Help seeking behavior |
| S29 | S18 OR S19 OR S20 OR S21 OR S22 OR S23 OR S24 OR S25 OR S26 OR S27 OR S28 |
| S30 | TI “partner violence” OR AB “partner violence” |
| S31 | TI “partner abuse” OR AB “partner abuse” |
| S32 | TI “relationship violence” OR AB “relationship violence” |
| S33 | TI “couple violence” OR AB “couple violence” |
| S34 | TI “dating violence” OR AB “dating violence” |
| S35 | TI “domestic violence” OR AB “domestic violence” |
| S36 | TI “family violence” OR AB “family violence” |
| S37 | TI “domestic abuse” OR AB “domestic abuse” |
| S38 | TI IPV OR AB IPV |
| S39 | TI “marital violence” OR AB “marital violence” |
| S40 | TI “battered wom?n” OR AB “battered wom?n” |
| S41 | TI “battered female*” OR AB “battered female*” |
| S42 | TI “spous* violence” OR AB “spous* violence” |
| S43 | TI “spous* abuse” OR AB “spous* abuse” |
| S44 | TI “wife abuse” OR AB “wife abuse” |
| S45 | TI “wife beat*” OR AB “wife beat*” |
| S46 | TI "wife batter*" OR AB "wife batter*" |
| S47 | DE Intimate Partner Violence |
| S48 | DE Domestic Violence |
| S49 | S30 OR S31 OR S32 OR S33 OR S34 OR S35 OR S36 OR S37 OR S38 OR S39 OR S40 OR S41 OR S42 OR S43 OR S44 OR S45 OR S46 OR S47 OR S48 |
| S50 | TI "ethnic minorit*"" or AB "ethnic minorit*" |
| S51 | TI ethnic* OR AB ethnic* |
| S52 | TI race OR AB race |
| S53 | TI racial OR AB racial |
| S54 | TI migrant* OR AB migrant* |
| S55 | TI immigrant* OR AB immigrant* |
| S56 | TI refugee* OR AB refugee* |
| S57 | TI “English as a second language” OR AB “English as a second language” |
| S58 | TI ESL OR AB ESL |
| S59 | TI foreign* OR AB foreign* |
| S60 | TI minorit* OR AB minorit* |
| S61 | TI divers* OR AB divers* |
| S62 | TI asylum or AB asylum |
| S63 | TI "mulitcultur* group" or AB "multicultur* group" |
| S64 | TI "cultur* group" or AB "cultur* group" |
| S65 | DE "Racial and Ethnic Groups" OR DE "African Cultural Groups" OR DE "Arabs" OR DE "Asians" OR DE "Blacks" OR DE "European Cultural Groups" OR DE "Indigenous Populations" OR DE "Latinos/Latinas" OR DE "Romanies" OR DE "Tribes" OR DE "Whites" |
| S66 | S49 OR S50 OR S51 OR S52 OR S53 OR S54 OR S55 OR S56 OR S57 OR S58 OR S59 OR S60 OR S61 OR S62 OR S63 OR S64 OR S65 |
| S67 | S17 AND S29 AND S49 AND S66 |
| **Limiters** | Peer-reviewed, English, Adulthood |
| **Definitions** | Ti – title, Ab – abstract, DE – exact subject heading |

**CINAHL**

| S1 | TI norm* or AB norm* |
| --- | --- |
| S2 | TI stigma or AB stigma |
| S3 | TI attitud* or AB attitud* |
| S4 | TI belief* or AB belief* |
| S5 | TI tradition* or AB tradition* |
| S6 | TI custom* or AB custom* |
| S7 | TI value* or AB value* |
| S8 | TI trait* or AB trait* |
| S9 | TI stereotyp* or AB stereotyp* |
| S10 | TI prejudic* or AB prejudic* |
| S11 | TI factor* or AB factor* |
| S12 | TI "cultur* differen*" or AB "cultur* differen*" |
| S13 | TI "ethnic* differen*" or AB "ethnic* differen*" |
| S14 | DE Cultural Values |
| S15 | DE Social Values |
| S16 | DE Social Norms |
| S17 | DE Social Attitudes |
| S18 | S1 OR S2 OR S3 OR S4 OR S5 OR S6 OR S7 OR S8 OR S9 OR S10 OR S11 OR S12 OR S13 OR S14 OR S15 OR S16 OR S17 |
| S19 | TI help-seek* or AB help-seek* |
| S20 | TI "help seek*" or AB "help seek*" |
| S21 | TI "assistance seek*" or AB "assistance seek*" |
| S22 | TI "service access" or AB "Service access" |
| S23 | TI "service use" or AB "service use" |
| S24 | TI "utili?ation N2 service*" or AB "utili?ation N2 service*" |
| S25 | TI "utili? N2 service*" or AB "utili? N2 service*" |
| S26 | TI "engag* N2 service*" or AB "engag* N2 service*" |
| S27 | TI "information seek*" or AB "information seek*" |
| S28 | TI barrier* or AB barrier* |
| S29 | DE Help seeking behavior |
| S30 | S19 OR S20 OR S21 OR S22 OR S23 OR S24 OR S25 OR S26 OR S27 OR S28 OR S29 |
| S31 | TI “partner violence” OR AB “partner violence” |
| S32 | TI “partner abuse” OR AB “partner abuse” |
| S33 | TI “relationship violence” OR AB “relationship violence” |
| S34 | TI “couple violence” OR AB “couple violence” |
| S35 | TI “dating violence” OR AB “dating violence” |
| S36 | TI “domestic violence” OR AB “domestic violence” |
| S37 | TI “family violence” OR AB “family violence” |
| S38 | TI “domestic abuse” OR AB “domestic abuse” |
| S39 | TI IPV OR AB IPV |
| S40 | TI “marital violence” OR AB “marital violence” |
| S41 | TI “battered wom?n” OR AB “battered wom?n” |
| S42 | TI “battered female*” OR AB “battered female*” |
| S43 | TI “spous* violence” OR AB “spous* violence” |
| S44 | TI “spous* abuse” OR AB “spous* abuse” |
| S45 | TI “wife abuse” OR AB “wife abuse” |
| S46 | TI “wife beat*” OR AB “wife beat*” |
| S47 | TI "wife batter*" OR AB "wife batter*" |
| S48 | MH Intimate Partner Violence |
| S49 | MH Domestic Violence |
| S50 | S31 OR S32 OR S33 OR S34 OR S35 OR S36 OR S37 OR S38 OR S39 OR S40 OR S41 OR S42 OR S43 OR S44 OR S45 OR S46 OR S47 OR S48 OR S49 |
| S51 | TI "ethnic minorit*"" or AB "ethnic minorit*" |
| S52 | TI ethnic* OR AB ethnic* |
| S53 | TI race OR AB race |
| S54 | TI racial OR AB racial |
| S55 | TI migrant* OR AB migrant* |
| S56 | TI immigrant* OR AB immigrant* |
| S57 | TI refugee* OR AB refugee* |
| S58 | TI “English as a second language” OR AB “English as a second language” |
| S59 | TI ESL OR AB ESL |
| S60 | TI foreign* OR AB foreign* |
| S61 | TI minorit* OR AB minorit* |
| S62 | TI divers* OR AB divers* |
| S63 | TI asylum or AB asylum |
| S64 | TI "mulitcultur* group" or AB "multicultur* group" |
| S65 | TI "cultur* group" or AB "cultur* group" |
| S66 | MH Ethnic Groups |
| S67 | S51 OR S52 OR S53 OR S54 OR S55 OR S56 OR S57 OR S58 OR S59 OR S60 OR S61 OR S62 OR S63 OR S64 OR S65 OR S66 |
| S68 | S18 AND S30 AND S50 AND S67 |
| **Limiters** | Peer-reviewed, English, Adulthood |
| **Definitions** | Ti – title, Ab – abstract, DE – exact subject heading, MH – broader subject heading |

**Global Health**

| S1 | TI norm* or AB norm* |
| --- | --- |
| S2 | TI stigma or AB stigma |
| S3 | TI attitud* or AB attitud* |
| S4 | TI belief* or AB belief* |
| S5 | TI tradition* or AB tradition* |
| S6 | TI custom* or AB custom* |
| S7 | TI value* or AB value* |
| S8 | TI trait* or AB trait* |
| S9 | TI stereotyp* or AB stereotyp* |
| S10 | TI prejudic* or AB prejudic* |
| S11 | TI factor* or AB factor* |
| S12 | TI "cultur* differen*" or AB "cultur* differen*" |
| S13 | TI "ethnic* differen*" or AB "ethnic* differen*" |
| S14 | DE Cultural Values |
| S15 | DE Social Values |
| S16 | S1 OR S2 OR S3 OR S4 OR S5 OR S6 OR S7 OR S8 OR S9 OR S10 OR S11 OR S12 OR S13 OR S14 OR S15 OR S16 |
| S17 | TI help-seek* or AB help-seek* |
| S18 | TI "help seek*" or AB "help seek*" |
| S19 | TI "assistance seek*" or AB "assistance seek*" |
| S20 | TI "service access" or AB "Service access" |
| S21 | TI "service use" or AB "service use" |
| S22 | TI "utili?ation N2 service*" or AB "utili?ation N2 service*" |
| S23 | TI "utili? N2 service*" or AB "utili? N2 service*" |
| S24 | TI "engag* N2 service*" or AB "engag* N2 service*" |
| S25 | TI "information seek*" or AB "information seek*" |
| S26 | TI barrier* or AB barrier* |
| S27 | S17 OR S18 OR S19 OR S20 OR S21 OR S22 OR S23 OR S24 OR S25 OR S26 |
| S28 | TI “partner violence” OR AB “partner violence” |
| S29 | TI “partner abuse” OR AB “partner abuse” |
| S30 | TI “relationship violence” OR AB “relationship violence” |
| S31 | TI “couple violence” OR AB “couple violence” |
| S32 | TI “dating violence” OR AB “dating violence” |
| S33 | TI “domestic violence” OR AB “domestic violence” |
| S34 | TI “family violence” OR AB “family violence” |
| S35 | TI “domestic abuse” OR AB “domestic abuse” |
| S36 | TI IPV OR AB IPV |
| S37 | TI “marital violence” OR AB “marital violence” |
| S38 | TI “battered wom?n” OR AB “battered wom?n” |
| S39 | TI “battered female*” OR AB “battered female*” |
| S40 | TI “spous* violence” OR AB “spous* violence” |
| S41 | TI “spous* abuse” OR AB “spous* abuse” |
| S42 | TI “wife abuse” OR AB “wife abuse” |
| S43 | TI “wife beat*” OR AB “wife beat*” |
| S44 | TI "wife batter*" OR AB "wife batter*" |
| S45 | DE "spouse abuse" |
| S46 | S28 OR S29 OR S30 OR S31 OR S32 OR S33 OR S34 OR S35 OR S36 OR S37 OR S38 OR S39 OR S40 OR S41 OR S42 OR S43 OR S44 OR S45 |
| S47 | TI "ethnic minorit*"" or AB "ethnic minorit*" |
| S48 | TI ethnic* OR AB ethnic* |
| S49 | TI race OR AB race |
| S50 | TI racial OR AB racial |
| S51 | TI migrant* OR AB migrant* |
| S52 | TI immigrant* OR AB immigrant* |
| S53 | TI refugee* OR AB refugee* |
| S54 | TI “English as a second language” OR AB “English as a second language” |
| S55 | TI ESL OR AB ESL |
| S56 | TI foreign* OR AB foreign* |
| S57 | TI minorit* OR AB minorit* |
| S58 | TI divers* OR AB divers* |
| S59 | TI asylum or AB asylum |
| S60 | TI "mulitcultur* group" or AB "multicultur* group" |
| S61 | TI "cultur* group" or AB "cultur* group" |
| S62 | DE "ethnic groups" |
| S63 | S47 OR S48 OR S49 OR S50 OR S51 OR S52 OR S53 OR S54 OR S55 OR S56 OR S57 OR S58 OR S59 OR S60 OR S61 OR S62 |
| S64 | S16 AND S27 AND S46 AND S63 |
| **Limiters** | English, Adulthood |
| **Definitions** | Ti – title, Ab – abstract, DE – exact subject heading, MH – broader subject heading |

**SocINDEX**

| S1 | TI norm* or AB norm* |
| --- | --- |
| S2 | TI stigma or AB stigma |
| S3 | TI attitud* or AB attitud* |
| S4 | TI belief* or AB belief* |
| S5 | TI tradition* or AB tradition* |
| S6 | TI custom* or AB custom* |
| S7 | TI value* or AB value* |
| S8 | TI trait* or AB trait* |
| S9 | TI stereotyp* or AB stereotyp* |
| S10 | TI prejudic* or AB prejudic* |
| S11 | TI factor* or AB factor* |
| S12 | TI "cultur* differen*" or AB "cultur* differen*" |
| S13 | TI "ethnic* differen*" or AB "ethnic* differen*" |
| S14 | DE Cultural Values |
| S15 | DE Social Values |
| S16 | DE Social Norms |
| S17 | DE Attitudes of Ethnic Groups |
| S18 | S1 OR S2 OR S3 OR S4 OR S5 OR S6 OR S7 OR S8 OR S9 OR S10 OR S11 OR S12 OR S13 OR S14 OR S15 OR S16 OR S17 |
| S19 | TI help-seek* or AB help-seek* |
| S20 | TI "help seek*" or AB "help seek*" |
| S21 | TI "assistance seek*" or AB "assistance seek*" |
| S22 | TI "service access" or AB "Service access" |
| S23 | TI "service use" or AB "service use" |
| S24 | TI "utili?ation N2 service*" or AB "utili?ation N2 service*" |
| S25 | TI "utili? N2 service*" or AB "utili? N2 service*" |
| S26 | TI "engag* N2 service*" or AB "engag* N2 service*" |
| S27 | TI "information seek*" or AB "information seek*" |
| S28 | TI barrier* or AB barrier* |
| S29 | DE "HELP-Seeking behavior" |
| S30 | DE "Utilisation of community mental health services" |
| S31 | S19 OR S20 OR S21 OR S22 OR S23 OR S24 OR S25 OR S26 OR S27 OR S28 OR S29 OR S30 |
| S32 | TI “partner violence” OR AB “partner violence” |
| S33 | TI “partner abuse” OR AB “partner abuse” |
| S34 | TI “relationship violence” OR AB “relationship violence” |
| S35 | TI “couple violence” OR AB “couple violence” |
| S36 | TI “dating violence” OR AB “dating violence” |
| S37 | TI “domestic violence” OR AB “domestic violence” |
| S38 | TI “family violence” OR AB “family violence” |
| S39 | TI “domestic abuse” OR AB “domestic abuse” |
| S40 | TI IPV OR AB IPV |
| S41 | TI “marital violence” OR AB “marital violence” |
| S42 | TI “battered wom?n” OR AB “battered wom?n” |
| S43 | TI “battered female*” OR AB “battered female*” |
| S44 | TI “spous* violence” OR AB “spous* violence” |
| S45 | TI “spous* abuse” OR AB “spous* abuse” |
| S46 | TI “wife abuse” OR AB “wife abuse” |
| S47 | TI “wife beat*” OR AB “wife beat*” |
| S48 | TI "wife batter*" OR AB "wife batter*" |
| S49 | DE "INTIMATE partner violence" OR DE "DATING violence" OR DE "MARITAL violence" |
| S50 | DE "domestic violence" |
| S51 | S32 OR S33 OR S34 OR S35 OR S36 OR S37 OR S38 OR S39 OR S40 OR S41 OR S42 OR S43 OR S44 OR S45 OR S46 OR S47 OR S48 OR S49 OR S50 |
| S52 | TI "ethnic minorit*"" or AB "ethnic minorit*" |
| S53 | TI ethnic* OR AB ethnic* |
| S54 | TI race OR AB race |
| S55 | TI racial OR AB racial |
| S56 | TI migrant* OR AB migrant* |
| S57 | TI immigrant* OR AB immigrant* |
| S58 | TI refugee* OR AB refugee* |
| S59 | TI “English as a second language” OR AB “English as a second language” |
| S60 | TI ESL OR AB ESL |
| S61 | TI foreign* OR AB foreign* |
| S62 | TI minorit* OR AB minorit* |
| S63 | TI divers* OR AB divers* |
| S64 | TI asylum or AB asylum |
| S65 | TI "mulitcultur* group" or AB "multicultur* group" |
| S66 | TI "cultur* group" or AB "cultur* group" |
| S67 | DE "ETHNIC groups" OR DE "AFRICAN Americans" OR DE "ARABS" OR DE "ARCTIC peoples" OR DE "ASIAN Americans" OR DE "ASIANS" OR DE "ETHNIC groups in mass media" OR DE "ETHNIC relations" OR DE "ETHNOLINGUISTIC groups" OR DE "EUROPEANS" OR DE "HISPANIC Americans" OR DE "INDIGENOUS peoples" OR DE "INDIGENOUS peoples of the Americas" OR DE "RACIALLY mixed people" |
| S68 | DE "RACIAL minorities" |
| S69 | S52 OR S53 OR S54 OR S55 OR S56 OR S57 OR S58 OR S59 OR S60 OR S61 OR S62 OR S63 OR S64 OR S65 OR S66 OR S67 OR S68 |
| S70 | S18 AND S31 AND S51 AND S69 |
| **Limiters** | English, Adulthood, Peer-reviewed |
| **Definitions** | Ti – title, Ab – abstract, DE – exact subject heading, MH – broader subject heading |
