# Supplementary material for: Influence of Cultural Norms on Formal Service Engagement Among Survivors of Intimate Partner Violence: A Qualitative Meta-synthesis
Source: Trauma Violence Abuse. 2023 Apr 19;25(1):738–51. doi: 10.1177/15248380231162971 (PMC10666477; doi:10.1177/15248380231162971)
Supplement: sj-docx-2-tva-10.1177_15248380231162971 – Supplemental material for Influence of Cultural Norms on Formal Service Engagement Among Survivors of Intimate Partner Violence: A Qualitative Meta-synthesis [file sj-docx-2-tva-10.1177_15248380231162971.docx]

**Appendix B**

*Quality Assessment of Included Studies*

| Reference (Year) |  |  |  |  | Criteria |  |  |  |  |  | Overall assessment of methodological quality |
| --- | --- | --- | --- | --- | --- | --- | --- | --- | --- | --- | --- |
|  | 1 | 2 | 3 | 4 | 5 | 6 | 7 | 8 | 9 | 10 |  |
| Acevedo (2000) | + | + | + | + | + | ? | ? | + | + | + | No or very minor concerns |
| Ahmad et al. (2009) | + | + | + | + | + | + | + | + | + | + | No or very minor concerns |
| Bauer et al. (2000) | + | + | + | ? | + | ? | ? | + | ? | ? | Minor concerns |
| Bhuyan et al. (2005) | + | + | + | + | + | + | + | + | + | + | No or very minor concerns |
| Briones-Vozmediano et al. (2019) | + | + | + | + | + | - | + | + | + | + | No or very minor concerns |
| Bui (2003) | + | + | + | + | + | - | + | ? | + | + | Minor concerns |
| Bui & Morash (2007) | ? | + | + | + | + | + | + | - | + | + | Minor concerns |
| Erez & Globokar (2009) | ? | + | + | ? | ? | ? | - | - | ? | + | Moderate concerns |
| Falconier et al. (2013) | + | + | + | + | + | + | - | + | + | + | No or very minor concerns |
| Femi-Ajao (2018) | + | + | + | + | + | + | + | + | + | + | No or very minor concerns |
| Gonzalez-Guarda et al. (2016) | + | + | + | + | + | ? | + | + | + | + | No or very minor concerns |
| Guruge & Humphreys (2009) | + | + | + | + | + | ? | - | ? | + | + | Minor concerns |
| Hassan & Cankurtaran (2021) | + | + | + | - | + | ? | + | ? | + | + |  |
| Kasturirangan & Nutt-Williams (2003) | + | + | + | + | + | + | + | + | + | + | No or very minor concerns |
| Keller & Brennan (2007) | + | + | + | + | + | - | - | ? | + | + | Minor concerns |
| Kelly (2009) | + | + | + | + | + | + | + | + | + | + | No or very minor concerns |
| Kulwicki et al. (2010) | + | + | + | + | + | - | ? | + | + | + | Minor concerns |
| Lewis et al. (2005) | + | + | + | + | + | ? | + | + | + | + | No or very minor concerns |
| Magnussen et al. (2011) | + | + | + | + | + | + | ? | + | + | ? | No or very minor concerns |
| McCleary-Sills et al. (2016) | + | + | + | + | + | ? | + | + | + | + | No or very minor concerns |
| Monterrosa (2019) | + | + | ? | + | + | - | + | + | + | + | Minor concerns |
| Mookerjee et al. (2015) | + | + | ? | + | ? | + | + | + | + | + | No or very minor concerns |
| Nicolaidis et al. (2010) | + | + | + | + | + | ? | - | + | + | + | Minor concerns |
| Park & Ko (2021) | + | + | + | + | + | - | + | + | + | + | No or very minor concerns |
| Raj & Silverman (2007) | + | + | ? | + | ? | - | + | + | + | + | Minor concerns |
| Reina et al. (2014) | + | + | + | + | + | ? | + | + | + | + | No or very minor concerns |
| Rodriguez et al. (1996) | + | + | + | + | + | ? | ? | + | + | + | No or very minor concerns |
| Rodriguez et al. (1998) | ? | + | ? | + | ? | - | + | + | + | + | Minor concerns |
| Sears (2021) | + | + | + | + | + | + | + | + | + | + | No or very minor concerns |
| Shen (2010) | + | + | + | + | + | ? | + | + | + | + | No or very minor concerns |
| Shirwadkar (2004) | ? | + | + | ? | + | + | - | - | + | + | Moderate concerns |
| Tam et al. (2016) | + | + | ? | ? | ? | - | + | ? | + | + | Moderate concerns |
| Thongpriwan et al. (2015) | + | + | + | + | + | - | + | + | + | + | No or very minor concerns |
| Ting (2010) | + | + | + | + | + | - | + | + | + | + | No or very minor concerns |
| Ting & Panchanadeswaran (2016) | + | + | + | ? | + | - | + | ? | + | + | Minor concerns |
| Tse (2007) | + | + | + | + | ? | ? | - | + | + | + | Minor concerns |
| Wolf et al. (2003) | + | + | + | + | + | ? | + | + | + | + | No or very minor concerns |
| Yang et al. (2022) | + | + | + | + | + | + | + | + | + | + | No or very minor concerns |

*Note.* **Criteria:** 1 = Was there a clear statement of the aims? 2 = Was a qualitative methodology appropriate? 3 = Was the research design appropriate? 4 = Was the recruitment strategy appropriate? 5 = Was the method of data collection appropriate? 6 = Was the relationship between the researcher and participant adequately considered? 7 = Were ethical issues taken into consideration? 8 = Was the data analysis sufficiently rigorous? 9= Was there a clear statement of the findings? 10 = Was the value of the research discussed? **Symbols:** + =yes; – = no;? = unclear.
