# Supplementary material for: Influence of Cultural Norms on Formal Service Engagement Among Survivors of Intimate Partner Violence: A Qualitative Meta-synthesis
Source: Trauma Violence Abuse. 2023 Apr 19;25(1):738–51. doi: 10.1177/15248380231162971 (PMC10666477; doi:10.1177/15248380231162971)
Supplement: sj-docx-3-tva-10.1177_15248380231162971 – Supplemental material for Influence of Cultural Norms on Formal Service Engagement Among Survivors of Intimate Partner Violence: A Qualitative Meta-synthesis [file sj-docx-3-tva-10.1177_15248380231162971.docx]

**Appendix C**

*Methodological Characteristics of Included Studies*

| Authors (Year) | Objective Summary  (To examine:) | Sample Size and Gender | Country | Participant Type | Methodology | Analysis method |
| --- | --- | --- | --- | --- | --- | --- |
| Acevedo (2000) | Perceptions and attitudes towards abuse and help-seeking behaviour among Mexican migrants. | 10 females | United States | Migrant victims of IPV | Individual interviews | Conceptual Analysis |
| Ahmad et al. (2009) | South Asian immigrant women’s reasons for delaying formal help-seeking. | 22 females | Canada | Migrant victims of IPV | 3 focus groups | Thematic Analysis |
| Bauer et al. (2000) | Social, political, and cultural barriers for Latina and Asian migrant women in accessing health services. | 28 females | United States | Migrant victims of IPV | 4 focus groups | Description consistent with Thematic Analysis |
| Bhuyan et al. (2005) | Cambodian women’s experiences, awareness and responses to DV, including cultural relevance of services. | 39 females | United States | Migrant victims of IPV | 6 focus groups | Description consistent with Thematic Analysis |
| Briones-Vozmediano et al. (2019) | Challenges and facilitators for detecting and addressing causes of IPV against Roma women. | 26 females  2 males | Spain | Primary care professionals and volunteer activists | Individual interviews | Thematic Analysis |
| Bui (2003) | Factors associated with migrant Vietnamese women’s decisions to seek help. | 34 females | United States | Migrant victims of IPV and service providers | Individual interviews | Not stated |
| Bui & Morash (2007) | Knowledge about abused Vietnamese women’s patterns of seeking help in a Vietnamese-American community, using a social capital framework. | 62 females | United States | Migrant victims of IPV | Individual interviews | Data were coded |
| Erez & Globokar (2009) | The challenges faced by abused immigrant women (from 35 countries of origin) in the United States and the cultural, linguistic and legal contexts within which they survive. | 137 female victims  40 service providers  26 legal representatives | United States | Migrant victims of IPV and service providers | Individual interviews | Not stated |
| Falconier et al. (2013) | Community perceptions of IPV, help-seeking behaviours, availability of services, and recommendations for a treatment program for Latina women. | 11 females and 6 males | United States | Health promoters and community members | 3 focus groups | Thematic Analysis |
| Femi-Ajao (2018) | Factors influencing disclosure and help-seeking among Nigerian women in England. | 16 females | England | Ethnic minority victims of IPV | Individual interviews | Thematic Analysis |
| Gonzalez-Guarda et al. (2016) | Dating violence and help-seeking experiences of Hispanic females in late adolescence living in Florida. | 11 females | United States | Ethnic minority victims of IPV | Individual interviews | Thematic Analysis |
| Guruge & Humphreys (2009) | Complexities of accessing and using formal social supports among immigrant Sri Lankan Tamil women living in Canada. | 10 females and 6 males | Canada | Community Leaders | Individual interviews | Thematic Analysis |
| Hassan & Cankurtaran (2022) | The reasons that prevent Syrian women refugees from seeking formal help. | 20 females | Turkey | Refugee victims of IPV | Individual interviews | Coded and analysed using MAXDA software |
| Kasturirangan & Williams (2009) | Latina survivors’ experience of IPV and approaches to enhance service providers’ cultural competence. | 9 females | United States | Non-Anglo-Saxon victims of IPV | Individual interviews | Consensual Qualitative Research Methodology |
| Keller & Brennan (2007) | The extent to which Sudanese immigrants adhere to traditional gender roles, and how cultural norms create barriers to service delivery. | 8 (gender not stated) | United States | Service providers | Individual interviews | Not stated |
| Kelly (2009) | The healthcare experiences of abused immigrant Latina mothers living in North America and their decision-making processes. | 17 females | United States | Migrant victims of IPV | 2 focus groups  and individual interviews | Thematic Analysis (Van Menen's approach) |
| Kulwicki et al. (2010) | Personal, socio-cultural, religious, legal and system barriers in utilisation of IPV services among Arab immigrant women | 65 (gender not stated) | United States | Community Leaders | 10 focus groups | Thematic analysis |
| Lewis et al. (2005) | Perceptions regarding IPV among the Latino community living in North America. | 53 females and 12 males | United States | Latino community members and service providers | Individual interviews | Deductive coding |
| Magnussen et al. (2011) | Interface between culture and IPV for women in 4 cultural groups in Hawaii. | 53 females | United States | Women accessing services affiliated with Community Health Centres. | 8 focus groups and individual interviews | Content Analysis |
| McCleary-Sills et al. (2016) | Sociocultural barriers that limit Tanzanian women’s agency in seeking support after experiencing IPV. | 104 (interviews – gender not stated)  48 females and 48 males (focus groups) | Tanzania | Service providers and duty bearers. | 12 participatory focus groups and individual interviews | Thematic Analysis workshops |
| Monterrosa (2019) | How internalized stereotypes affect the IPV-related help-seeking of African American women and white women living in Colorado. | 15 females | United States | Ethnic minority victims of IPV | Individual interviews | Theme analysis utilized Potter’s Black feminist criminology theory |
| Mookerjee et al. (2015) | Help-seeking behaviours between Hispanic and non-Hispanic IPV survivors. | 22 females | United States | Ethnic minority victims of IPV | 3 focus groups | Theme analysis utilized Liang et al.’s (2005) model of help-seeking |
| Nicolaidis et al. (2010) | Influence of racism, violence, and social context on African American women’s beliefs regarding depression and depression care. | 30 females | United States | Ethnic minority victims of IPV | 4 focus groups | Thematic Analysis |
| Park & Ko (2021) | South Korean female IPV victims’ experiences in seeking help based on their support selection. | 14 females | South Korea | Non-Anglo-Saxon victims of IPV | Individual interviews | Directed Content Analysis |
| Raj & Silverman (2002) | Help-seeking behaviours of South-Asian immigrant women in the United States. | 23 females | United States | Migrant victims of IPV | Individual interviews | Grounded Theory |
| Reina et al. (2014) | Latina immigrant victims’ experiences with IPV service outreach. | 10 females | United States | Migrant victims of IPV | 1 focus group and individual interviews | Qualitative data inquiry (Arcury & Quandt, 1998) |
| Rodriguez et al. (1996) | Common barriers to women of different ethnic groups (White, African American, Latina and Asian), living in California, that influence identification and management of IPV in the health care system. | 51 females | United States | Ethnic minority victims of IPV | 8 focus groups | Theme analysis using Ethnograph software |
| Rodriguez et al. (1998) | Health care provider-related factors that may affect patient-provider communication for Asian and Latina immigrant women. | 28 females | United States | Migrant victims of IPV | 4 focus groups | Theme analysis using Ethnograph software |
| Sears (2021) | The cultural beliefs and norms associated with the help-seeking decisions of abused Black Jamaican and African American women in the United States. | 21 females | United States | Ethnic minority victims of IPV | Individual interviews | Thematic Analysis |
| Shen (2011) | Help-seeking behaviours of female Taiwanese IPV victims. | 10 females | China | Non-Anglo-Saxon victims of IPV | Individual interviews | Thematic Analysis |
| Shirwadkar (2004) | Barriers to accessing Canadian policies and programs for Indian immigrant women. | 8 females | Canada | Victim advocates and activists and Indian immigrant women | Individual interviews | Case studies |
| Tam et al. (2016) | Challenges of ethnic minority women (Asian, mixed background, African/Caribbean and Hispanic) from three Canadian cities and their experiences with the police and criminal court’s response to IPV. | 14 females | Canada | Ethnic minority victims of IPV | Individual interviews | Thematic Analysis |
| Thongpriwan et al. (2015) | Perceptions of IPV among SEA migrant women attending college in the United States and how they recognize their vulnerability. | 18 females | United States | SEA young migrant women attending university | 3 focus groups | Content Analysis |
| Ting (2010) | Coping strategies utilized by immigrant African survivors of IPV living in the United States. | 15 females | United States | Migrant victims of IPV | Individual interviews | Constant Comparison method |
| Ting & Panchanadeswaran (2009) | Experiences and perceptions of help seeking with faith-based leaders and the role of spirituality among African immigrant women living in the United States. | 15 females | United States | Migrant victims of IPV | Individual interviews | Phenomenological Approach |
| Tse (2007) | IPV in Asian migrant communities in Aotearoa New Zealand. | 50 females and 6 males | New Zealand | Migrant victims and perpetrators of IPV | 1 focus group and individual interviews | Case Analysis and generation of themes and sub-themes |
| Wolf et al. (2003) | Perceptions of barriers and enablers to seeking police help for IPV among White, Asian, Native America and African American women living in the United States. | 41 females | United States | Ethnic minority women accessing social services | 5 focus groups | Coded and analysed themes using Ethnograph software |
| Yang Li et al. (2022) | Help-seeking experiences of abused Chinese immigrant women and barriers in accessing help in the United States. | 20 females | United States | Migrant victims of IPV | Individual interviews | Thematic Analysis |

*Note.* IPV = intimate partner violence. The term IPV is used for consistency, though studies may use alternative terms (e.g., domestic violence, partner abuse).
