# Supplementary material for: Influence of Cultural Norms on Formal Service Engagement Among Survivors of Intimate Partner Violence: A Qualitative Meta-synthesis
Source: Trauma Violence Abuse. 2023 Apr 19;25(1):738–51. doi: 10.1177/15248380231162971 (PMC10666477; doi:10.1177/15248380231162971)
Supplement: sj-docx-4-tva-10.1177_15248380231162971 – Supplemental material for Influence of Cultural Norms on Formal Service Engagement Among Survivors of Intimate Partner Violence: A Qualitative Meta-synthesis [file sj-docx-4-tva-10.1177_15248380231162971.docx]

**Appendix D**

*Themes and Subthemes Derived from the Included Studies*

| Theme/Subtheme | Description | No. of articles | Article Citations |
| --- | --- | --- | --- |
| 1. Gender Roles and Social Expectations | | | |
| Patriarchal beliefs | Beliefs of male dominance and female subordination normalized the violence and prevented identification of the abuse. | 21 | Acevedo (2000); Ahmad et al. (2009); Bauer et al. (2000); Bhuyan et al. (2005); Briones-Vozmediano et al. (2019); Bui (2003); Gonzalez-Guarda et al. (2016); Hassan & Cankurtaran (2022); Kasturirangan & Nutt-Williams (2003); Keller & Brennan (2007); Kulwicki et al. (2010); Lewis et al. (2005); Magnussen et al. (2011); McCleary-Sills et al. (2016); Reina et al. (2014); Rodriguez et al. (1996); Shen (2011); Thongpriwan et al. (2015); Ting (2010); Ting & Panchanadeswaran (2016); Tse (2007). |
| Marital roles and obligations | Beliefs about what is expected of men and women within a marriage often reinforced male dominance and female subordination. Divorce and pre-marital sex taboos and pressure to maintain family harmony deterred women from leaving the relationship. | 25 | Acevedo (2000); Ahmad et al. (2009); Bauer et al. (2000); Bhuyan et al. (2005); Briones-Vozmediano et al. (2019); Bui (2003); Femi-Ajao (2018); Guruge & Humphreys (2009); Hassan & Cankurtaran (2022); Kasturirangan & Nutt-Williams (2003); Keller & Brennan (2007); Kelly (2009); Kulwicki et al. (2010); McCleary-Sills et al. (2016); Park & Ko (2021); Raj & Silverman, (2007); Reina et al. (2014); Rodriguez et al. (1996); Shen (2011); Shirwadkar (2004); Tam et al. (2016); Thongpriwan et al. (2015); Ting (2010); Ting & Panchanadeswaran (2016); Tse (2007). |
| Self-sacrificing women | Expectations of women to tolerate the abuse for the benefit of others (children, the perpetrator, the broader community) resulted in silencing women. | 26 | Acevedo (2000); Ahmad et al. (2009); Bauer et al. (2000); Bhuyan et al. (2005); Briones-Vozmediano et al. (2019); Bui & Morash (2007); Bui (2003); Erez & Globokar (2009); Falconier et al. (2013); Hassan & Cankurtaran (2022); Kasturirangan & Nutt-Williams (2003); Keller & Brennan (2007); Kelly (2009); Kulwicki et al. (2010); Lewis et al. (2005); Magnussen et al. (2011); McCleary-Sills et al. (2016); Monterrosa (2019); Raj & Silverman (2007); Rodriguez et al. (1996); Sears (2021); Tam et al. (2016); Thongpriwan et al. (2015); Ting (2010); Ting & Panchanadeswaran (2016); Tse (2007). |
| 1. Community Recognition and Acceptance of Abuse | | | |
| Normalisation of abuse | Abuse was responded to as 'normal' which impacted the ability of women and the community to recognize the abuse and then identify the need for help. | 21 | Acevedo (2000); Ahmad et al. (2009); Bauer et al. (2000); Bhuyan et al. (2005); Briones-Vozmediano et al. (2019); Bui (2003); Falconier et al. (2013); Femi-Ajao (2018); Gonzalez-Guarda et al. (2016); Hassan & Cankurtaran (2022); Kasturirangan & Nutt-Williams (2003); Keller & Brennan (2007); Kulwicki et al. (2010); Lewis et al. (2005); McCleary-Sills et al. (2016); Monterrosa (2019); Raj & Silverman, (2007); Reina et al. (2014); Sears (2021); Thongpriwan et al. (2015); Ting (2010); Tse (2007). |
| Community acceptance moderated by severity | Abuse was accepted as normal until it increased in severity, at which point it was considered unacceptable and warranting formal intervention. | 7 | Acevedo (2000); Ahmad et al. (2009); Bui & Morash (2007); McCleary-Sills et al. (2016); Rodriguez et al. (1996); Tam et al. (2016); Wolf et al. (2003). |
| 1. Honor-Based Society | | | |
| Defending the collective | The expectation that women will protect the community reputation at all costs, which involves staying silent. Also not involving 'outsiders' reduced women’s willingness to seek formal support. | 28 | Acevedo (2000); Ahmad et al. (2009); Bauer et al. (2000); Bhuyan et al. (2005); Briones-Vozmediano et al. (2019); Bui & Morash (2007); Bui (2003); Erez & Globokar (2009); Falconier et al. (2013); Guruge & Humphreys (2009); Kasturirangan & Nutt-Williams (2003); Keller & Brennan (2007); Kulwicki et al. (2010); Lewis et al. (2005); Magnussen et al. (2011); McCleary-Sills et al. (2016); Mookerjee et al. (2015); Park & Ko (2021); Raj & Silverman (2007); Reina et al. (2014); Rodriguez et al. (1996); Sears (2021); Shen (2011); Shirwadkar (2004); Thongpriwan et al. (2015); Ting & Panchanadeswaran (2016); Tse (2007); Yang Li et al. (2022). |
| Collective as protective | Qualities of collectivist cultures, such as communal living and involvement of the family and community in selecting partners, provide women with a level of protection, including a place to stay and the perpetrator being held to account by extended family/friends. | 6 | Ahmad et al. (2009); Guruge & Humphreys (2009); Magnussen et al. (2011); Raj & Silverman (2007); Sears (2021), Ting (2010). |
| 1. Role of Religion | | | |
| Religious beliefs as a coping mechanism | Prayer, faith, and belief in karma/fate provided women with a sense of hope that there would be justice, which helped women to endure the suffering but also muted the need for seeking further assistance. | 11 | Acevedo (2000); Ahmad et al. (2009); Bhuyan et al. (2005); Bui & Morash (2007); Magnussen et al. (2011); Sears (2021); Shen (2011); Ting (2010); Ting & Panchanadeswaran (2016); Tse (2007); Ying Li et al. (2022). |
| Influence of religious leaders | Religious leaders may provide emotional and practical support and assist in holding perpetrators to account. However, they reinforce cultural norms that silence women or normalize the violence. | 13 | *Positive influence:* Acevedo (2000); Bui & Morash (2007); Falconier et al. (2013); Femi-Ajao (2018); Magnussen et al. (2011); Ting (2010); Ying Li et al. (2022).  *Negative influence:* Acevedo (2000); Bui & Morash (2007); Bui (2003); Erez & Globokar (2009); Hassan & Cankurtaran (2022); Kulwicki et al. (2010); Shen (2011); Ting & Panchanadeswaran (2016). |
| 1. Cultural Beliefs and Attitudes Toward Formal Services | | | |
| IPV must be physical or severe | Perceptions (or previous experiences) that formal services should only be used if the violence is physical (or extremely severe). | 14 | Acevedo (2000); Ahmad et al. (2009); Briones-Vozmediano et al. (2019), Bui & Morash (2007); Bui (2003); Erez & Globokar (2009); Falconier et al. (2013); Kasturirangan & Nutt-Williams (2003); Kelly (2009); McCleary-Sills et al. (2016); Mookerjee et al. (2015); Park & Ko (2021); Raj & Silverman (2007); Wolf et al. (2003). |
| Formal services are a last resort | The belief that services should not be accessed unless other options have been exhausted or the violence has escalated beyond that that the community and individual can cope with. | 13 | Ahmad et al. (2009); Bhuyan et al. (2005); Bui (2003); Bui & Morash (2007); Falconier et al. (2013); Kasturirangan & Nutt-Williams (2003); Kelly (2009); Lewis et al. (2005); McCleary-Sills et al. (2016); Park & Ko (2021); Raj & Silverman (2007); Tam et al. (2015); Wolf et al. (2003). |
| Victims fear consequence of service engagement | Women feared the repercussions of seeking help including deportation, escalation in abuse, retribution from family members, involvement of child protection. | 23 | Acevedo (2000); Ahmad et al. (2009); Bauer et al. (2000); Bhuyan et al. (2005); Briones-Vozmediano et al. (2019); Bui & Morash (2007); Bui (2003); Erez & Globokar (2009); Femi-Ajao (2018); Hassan & Cankurtaran (2022); Kelly (2009); Lewis et al. (2005); Magnussen et al. (2011); McCleary-Sills et al. (2016); Monterrosa (2019); Mookerjee et al. (2015); Nicolaidis et al. (2015); Park & Ko (2021); Reina et al. (2014); Shen (2011); Shirwadkar (2004); Tam et al. (2016); Wolf et al. (2003). |
| Women do not have equal rights or access to services | Many women from culturally diverse communities are not aware that the services exist, what they do, how to access them, or their legal rights to access. | 15 | Acevedo (2000); Ahmad et al. (2009); Bhuyan et al. (2005); Bui & Morash (2007); Erez & Globokar (2009), Falconier et al. (2013); Gonzalez-Guarda et al. (2016); Guruge & Humphreys (2009); Kulwicki et al. (2010); Raj & Silverman (2007); Reina et al. (2014); Shen (2011); Tam et al. (2016); Ting (2010); Tse (2007). |
| Service responses penalize women | Women experienced or perceived service responses to be ineffective and penalising towards victims. These responses included delayed responses, inappropriate responses, blaming females; and mandatory reporting laws and policies that required women to report violence as a prerequisite to accessing support. | 24 | *Ineffective responses:* Acevedo (2000); Bauer et al. (2000); Briones-Vozmediano et al. (2019); Erez & Globokar (2009); Lewis et al. (2005); McCleary-Sills et al. (2016); Monterrosa (2019); Mookerjee et al. (2015); Nicolaidis et al. (2010); Park & Ko (2021); Reina et al. (2014); Rodriguez et al. (1998); Shen (2011); Shirwadkar (2004); Tam et al. (2016); Wolf et al. (2003).  *Laws and practices that penalize women:* Bauer et al. (2000); Bhuyan et al. (2005); Bui (2003); Erez & Globokar (2009); Femi-Ajao (2018); Gonzalez-Guarda et al. (2016); Kelly (2009); Kulwicki et al. (2010); Mookerjee et al. (2015); Reina et al. (2014); Shirwadkar (2004); Tam et al. (2016); Ting (2010); Tse (2007); Wolf et al. (2003) |
| Services are not culturally relevant | Belief that services are racist, linguistically inaccessible, or not culturally sensitive and relevant. | 24 | Bauer et al. (2000); Bhuyan et al. (2005); Briones-Vozmediano et al. (2019); Erez & Globokar (2009); Falconier et al. (2013); Femi-Ajao (2018); Guruge & Humphreys (2009); Kasturirangan & Nutt-Williams (2003); Keller & Brennan (2007); Kulwicki et al. (2010); Monterrosa (2019); Mookerjee et al. (2015); Nicolaidis et al. (2015); Park & Ko (2021); Raj & Silverman (2007); Reina et al. (2014); Rodriguez et al. (1996); Rodriguez et al. (1998); Shirwadkar (2004); Tam et al. (2016); Thongpriwan et al. (2015); Tse (2007); Wolf et al. (2003); Yang Li et al. (2022). |
